# Restoration of Autophagy and Apoptosis in Myelodysplastic Syndromes: The Effect of Azacitidine in Disease Pathogenesis

**DOI:** 10.3390/cimb47070520

**Published:** 2025-07-04

**Authors:** Georgia Tsekoura, Andreas Agathangelidis, Christina-Nefeli Kontandreopoulou, Eirini Sofia Fasouli, Eleni Katsantoni, Vaia Pliaka, Leonidas Alexopoulos, Eleni Katana, Myrto Papaioannou, Georgia Taktikou, Maria Eleftheria Strataki, Angeliki Taliouraki, Marina Mantzourani, Nora-Athina Viniou, Panagiotis T. Diamantopoulos, Panagoula Kollia

**Affiliations:** 1 Division of Genetics & Biotechnology, Department of Biology, National and Kapodistrian University of Athens, 15772 Athens, Greece; www.tsekou@yahoo.gr (G.T.); elekatana@biol.uoa.gr (E.K.); gtaktikou@gmail.com (G.T.); mariliastrataki24@gmail.com (M.E.S.);; 2 First Department of Internal Medicine, National and Kapodistrian University of Athens, 11527 Athens, Greece; nefknt@med.uoa.gr (C.-N.K.); jbnv@otenet.gr (N.-A.V.); diamp@med.uoa.gr (P.T.D.); 3 Basic Research Center, Biomedical Research Foundation, Academy of Athens, 11527 Athens, Greece; esfasouli@bioacademy.gr (E.S.F.); ekatsantoni@bioacademy.gr (E.K.); 4 Protavio Ltd., Demokritos Science Park, 15341 Athens, Greece; vicky.pliaka@protavio.com (V.P.); leo@protatonce.com (L.A.); 5 Department of Mechanical Engineering, National Technical University of Athens, 15773 Athens, Greece; 6 Department of Biomedical Sciences, School of Health Sciences, University of West Attica, 12243 Athens, Greece; myrtop@yahoo.com

**Keywords:** myelodysplastic syndromes, autophagy, apoptosis, protein expression profiling, azacitidine

## Abstract

Myelodysplastic syndromes (MDSs) comprise a diverse group of clonal hematopoietic stem cell disorders characterized by ineffective hematopoiesis, cytopenia in the peripheral blood, and an increased risk of transformation into acute myeloid leukemia (AML). Despite extensive research, the mechanisms underlying MDS pathogenesis remain unclear. In the present study, we explored the role of autophagy and apoptosis in the development of MDS and assessed the impact of azacitidine on these processes in vitro. First, we assessed the expression of proteins involved in both autophagic and apoptotic pathways in MDS patients with different prognoses. Furthermore, using the MDS-L cell line as a model, we investigated the in vitro effects of azacitidine treatment on these processes. We report that MDS, irrespective of risk classification, is associated with the dysregulation of autophagy and apoptosis. Notably, azacitidine treatment restored these cellular processes, accompanied by modulation of key signaling phosphoproteins. Overall, these findings provide evidence that impaired autophagy and apoptosis contribute to MDS pathogenesis and that azacitidine helps restore cellular homeostasis by activating both processes. Furthermore, our study highlights the potential therapeutic benefits of targeting these mechanisms and suggests that combining azacitidine with agents that modulate autophagy and apoptosis could enhance the treatment efficacy for MDS patients.

## 1. Introduction

Myelodysplastic syndromes (MDSs) are clonal hematological malignancies characterized by ineffective hematopoiesis, cytopenia, and an increased risk of progression to acute myeloid leukemia (AML) [1]. MDS predominantly affects the elderly, with an incidence of approximately 20 per 100,000 individuals [2]. Despite extensive research, the mechanisms underlying MDS pathogenesis remain unclear. A prominent feature of MDS is the dysregulation of fundamental cellular processes, particularly autophagy [3] and apoptosis [4].

Autophagy is closely associated with MDS development. In this context, impairment of the PI3K/AKT pathway often results in cytopenia, dysplasia [5], and anemia in patients with MDS [6]. Furthermore, certain autophagy genes serve as prognostic markers, categorizing patients into high- and low-risk groups [7]. Apoptosis plays a dual role in MDS: it contributes to ineffective hematopoiesis in the early stages, while its inhibition is associated with disease progression [8]. As MDS advances, increased expression of anti-apoptotic proteins like BCL2 is observed, which can facilitate transformation into AML [9]. Consequently, therapeutic strategies that modulate apoptosis, such as the use of the BCL2 inhibitor venetoclax, are under investigation in MDS treatment [10].

The interplay between autophagy and apoptosis is complex, with both processes exhibiting cooperative and antagonistic relationships in the context of cell death [11]. Recent evidence, including our own previous work, has shown that MDS patients exhibit significant downregulation of genes associated with both autophagy and apoptosis, with this effect being more pronounced effect in higher-risk patients [12], as defined by the IPSS-R scoring system [13].

Currently, the therapeutic management of MDS involves a range of treatment modalities. Among these, azacitidine is one of the most well-established disease-modifying drugs [14,15,16], shown to improve survival rates mostly in higher-risk patients [17]. However, the precise downstream effects of azacitidine on autophagy and apoptosis remain largely unknown, limiting our understanding of its full therapeutic potential and its impact on disease pathogenesis.

To address this issue, we assessed the expression of proteins involved in autophagy and apoptosis in MDS patients with different disease risks. Subsequently, using the MDS-L cell line as a model, we investigated the in vitro effects of azacitidine on these processes. We report that MDS, irrespective of risk classification, is associated with the dysregulation of autophagy and apoptosis. Notably, azacitidine treatment restored these cellular processes, accompanied by the modulation of key signaling phosphoproteins. In summary, our findings corroborate the notion that the impairment of apoptosis and autophagy is crucial to MDS pathogenesis, while their restoration using existing or novel therapeutic drugs could offer important therapeutic benefits to patients with MDS.

## 2. Materials and Methods

### 2.1. The Study Group

The study group comprised 20 untreated patients with MDS and 14 healthy donors. The MDS patients were divided into lower-risk (LR-MDS, n = 12) and higher-risk groups (HR-MDS, n = 8), according to the IPSS-R scoring system [13]. More information regarding the clinical and biological characteristics of the MDS patients is given in the Supplementary Materials and Supplementary Tables S1 and S2.

### 2.2. Primary Cell Separation from MDS Patients

Mononuclear cells were isolated using Ficoll–Paque medium (GE Healthcare, Bio-Sciences AB, Uppsala, Sweden) and pelleted after erythrocyte lysis and two washes in phosphate-buffered saline (PBS tablets) (Calbiochem, Merck KGaA, Darmstadt, Germany).

### 2.3. The MDS-L Cell Line Culture

The MDS-L cell line was provided by Dr. Kaoru Tohyama (Kawasaki Medical School, Okayama, Japan). The MDS-L cells were cultured in RPMI 1640 medium with 10% FBS, 1% penicillin–streptomycin, and 10 ng/mL human recombinant IL-3 (Stemcell Technologies, Vancouver, BC, Canada).

### 2.4. Azacitidine Preparation

Azacitidine Vidaza (25mg/mL) (Celgene Europe B.V., Utrecht, The Netherlands) was freshly prepared using DEPC water.

### 2.5. MDS-L Cell Viability After Azacitidine Treatment

The MDS-L cell line was cultured with different azacitidine concentrations (namely 0.5, 1, 3, and 5 μM) for various time intervals (namely 24, 48, and 72 h), and cell apoptosis was assessed. More information is provided in the Supplementary Materials.

### 2.6. RNA and Protein Extraction from the MDS-L Cells After Azacitidine Treatment

RNA and proteins were extracted from the MDS-L cell samples containing 10^6^ cells/mL after treatment with 0.5 μM of azacitidine for 48 h. Total RNA was extracted using TRIZOL (Invitrogen, Carlsbad, CA, USA). Proteins were extracted using a radioimmunoprecipitation assay buffer and quantified using a Bicinchoninic Acid (BCA) Protein Assay Kit (Thermo Fisher Scientific, Waltham, MA, USA).

### 2.7. The Gene Expression Analysis Using Quantitative Real-Time PCR

cDNA synthesis and PCR amplification were performed as described previously [12]. The expression of genes related to autophagy and apoptosis was assessed before and after azacitidine treatment. All experiments were run in triplicate. Supplementary Table S3 summarizes the 16 target genes and the respective primer sequences.

### 2.8. The Protein Expression Analysis Using Western Blotting

Proteins were separated and incubated with specific primary antibodies. Blots were probed with secondary goat anti-mouse or anti-rabbit antibodies and visualized. The analysis of the expression of relevant proteins was conducted in primary cells from the MDS patients, as well as in the MDS-L cells before and after azacitidine treatment. The experiments were performed three times for each sample in both analyses. Subsequently, the mean values were calculated, and the standard deviation (SD) or standard error (SE) was employed to indicate the data distribution. The presence of the GAPDH protein was assessed in all cases as a reference. Supplementary Table S4 summarizes all of the primary and secondary antibodies utilized.

### 2.9. The Phosphoprotein Expression Analysis Using a Multiplex ELISA Assay

The MDS-L cells were lysed, and phosphoprotein levels were assessed using a custom-developed phosphoprotein 21-plex panel (ProtATonce, Athens, Greece) (Supplementary Table S5). These experiments were performed in three treated and three untreated samples.

### 2.10. Statistical Approach

The data were analyzed using SPSS version 13.0 software (SPSS Inc., Chicago, IL, USA). The significance of the differences between groups was assessed using the Student’s *t*-test, while a one-way ANOVA and Tukey’s post hoc test were used for multiple comparisons. Statistical significance was defined as *p* < 0.05.

## 3. Results

### 3.1. MDS Is Characterized by the Downregulation of Autophagy and Apoptosis Proteins

To assess the role of autophagy in MDS pathogenesis, we analyzed the expression of 11 relevant autophagic proteins in all MDS groups compared to that in healthy donors (Table 1). The expression levels of the pro-apoptotic proteins CASP3, CASP7, and CASP8, as well as the BCL2 protein, were also investigated. The analysis was performed in the following groups: (a) healthy donors, (b) all MDS patients, (c) the LR-MDS group, and (d) the HR-MDS group; comparisons made performed between healthy donors and each MDS group.

Overall, we observed lower expression levels for most proteins (12/15, 80%) in the patients with MDS compared to those in the healthy controls (Table 1). In terms of autophagy, significantly lower levels of 3/11 proteins (27.3%), namely ATG5, CTSB, and LC3II, were exhibited in all of the MDS groups (namely all MDS patients, LR-MDS, and HR-MDS) versus those in healthy donors. In addition, ATG12 and DRAM1 exhibited the same expression pattern, yet their downregulation was significant only in the all MDS patient and HR-MDS groups. Finally, the downregulation of AMBRA1, ATG16, PI3KC3, and UVRAG in the MDS groups compared to healthy individuals was not significant. On the other hand, TGM2 and LC3I were upregulated in the MDS patients compared to healthy donors, which was significant only for TGM2.

**Figure 1:**
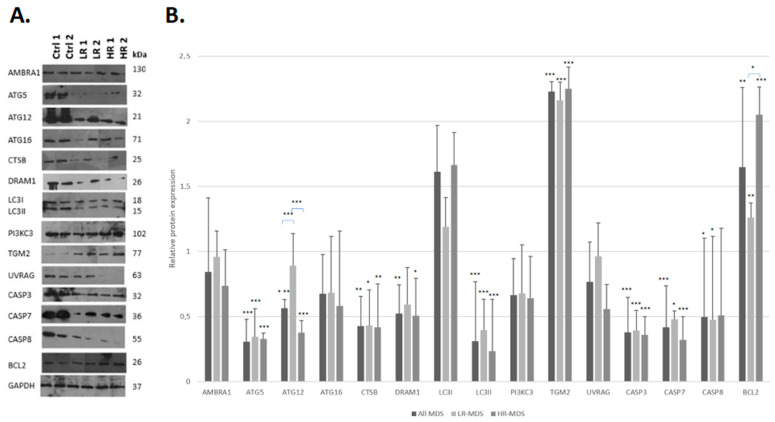
Lower expression levels of proteins implicated in the processes of autophagy and apoptosis in MDS patients compared to those in healthy donors. (**A**) Western blot experiments in representative samples from the groups of healthy donors (Ctrl1, Ctrl2) and MDS patients of different classifications (HR-MDS: HR1, HR2; LR-MDS: LR1, LR2). The intensity of the bands was quantified using the ImageJ 1.x software, while the expression ratio of each protein was estimated relative to that for the GAPDH protein. (**B**) Relative expression levels in the group of all MDS patients as well as in each of the MDS risk groups compared to those in the healthy controls. The statistical examination was performed using a one-way ANOVA and Tukey’s post hoc test. Asterisks represent statistically significant differences; one asterisk (*) corresponds to *p* < 0.05, two asterisks (**) correspond to *p* < 0.02, and three asterisks (***) correspond to *p*< 0.001. Error bars correspond to the standard deviation (SD).

At the level of apoptosis, significantly lower levels of all three caspases were found in all MDS groups versus those in healthy donors, with the exception of CASP8 in HR-MDS. In contrast, BCL2 was significantly upregulated in all of the MDS groups versus healthy donors. The protein expression levels in all MDS groups as well as the group of healthy donors are given in Figure 1.

Lastly, the comparisons among the MDS groups showed that both autophagy and apoptosis were downregulated in HR-MDS compared to LR-MDS, as evidenced by the lower expression levels for 9/11 autophagy-related proteins. In contrast, LC3I and TGM2 were upregulated in HR-MDS compared to LR-MDS. In terms of apoptosis, HR-MDS was characterized by lower expression levels of CASP3 and CASP7, as well as the significant upregulation of BCL2 (Supplementary Table S6).

### 3.2. Azacytidine Induces Effective Cell Apoptosis in the MDS-L Cell Line

**Figure 2:**
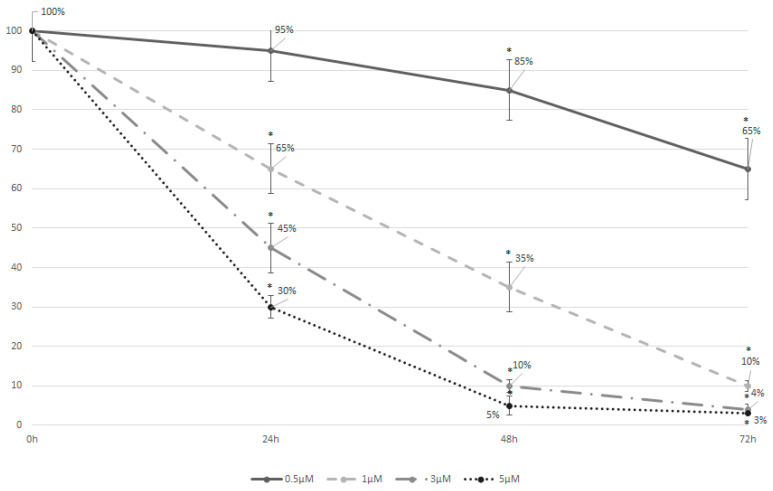
Treatment with azacitidine can induce strong cell apoptosis in MDS-L cells even after 24 h of culture. The graph depicts the cell viability curves for MDS-L cells treated with different concentrations of azacitidine (0.5 μΜ, 1 μΜ, 3 μΜ, or 5 μM) after 24, 48, and 72 h. Higher concentrations of azacitidine demonstrated an enhanced cytotoxic effect on the cells, with the degree of cytotoxicity progressively increasing over time, independent of the concentration. Asterisks represent statistically significant differences corresponding to *p* < 0.05. Error bars correspond to the standard errors (SEs).

Higher concentrations of azacitidine exhibited a stronger cytotoxic effect on the cells, with the extent of cytotoxicity increasing continuously over time irrespective of the concentration (Figure 2). In particular, azacitidine concentrations of 3 and 5 μM achieved an IC50 within only 24 h of treatment. On the other hand, a concentration of 0.5 μM was toxic to only 5% of the MDS-L cells after 24 h, reaching 15% and 35% at the timepoints of 48 h and 72 h, respectively. Based on these findings, all subsequent experiments were performed in MDS-L cells treated with 0.5 μM of azacitidine for 48 h.

### 3.3. Treatment of the MDS-L Cells with Azacitidine Restored Autophagy and Apoptosis at the mRNA and Protein Levels

**Figure 3:**
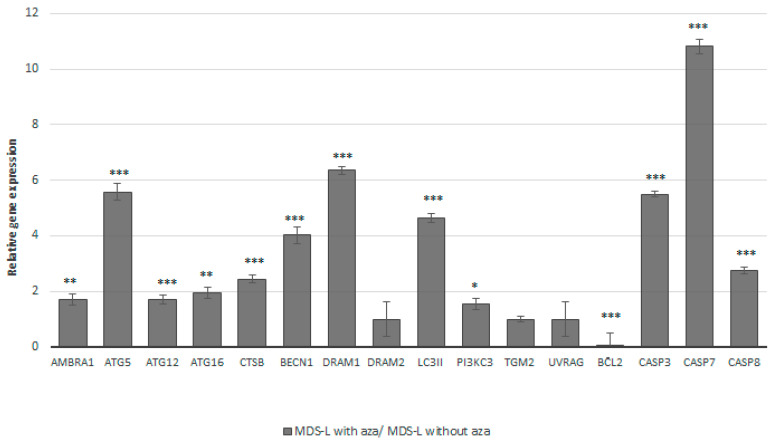
Treatment of MDS-L cells with a low concentration of azacitidine may lead to the restoration of autophagy and apoptosis. The expression patterns for a selected panel of 16 genes related to the processes of autophagy and apoptosis in MDS-L cells. Bars represent the relative gene expression levels in the MDS-L cells after treatment with azacitidine compared to those under the baseline status (i.e., without azacitidine treatment). Azacitidine treatment led to upregulation of all of the genes, besides downregulating the anti-autophagic and anti-apoptotic *BCL2* gene. The *HPRT1* gene was used as a reference. Asterisks represent statistically significant differences; one asterisk (*) corresponds to *p* < 0.05, two asterisks (**) correspond to *p* < 0.02, and three asterisks (***) correspond to *p*< 0.001. Error bars correspond to the standard errors (SEs).

Our gene expression analysis showed that the majority of the analyzed genes (12/16, 75%) exhibited a significant increase in their expression levels after azacitidine treatment. Specifically, we observed (i) a < 2-fold change in the autophagy genes *AMBRA1*, *ATG12*, *ATG16,* and *PI3KC3* and (ii) a > 2-fold change in the autophagy genes *ATG5*, *BECN1*, *CTSB*, *DRAM1*, and *LC3II* genes, as well as in the apoptotic genes *CASP3*, *CASP7,* and *CASP8*. The *BCL2* gene displayed a statistically significant 18-fold decrease in its expression after azacitidine treatment. Detailed information regarding the effect of azacitidine on the gene expression levels is given in Figure 3 and Supplementary Table S7.

**Figure 4:**
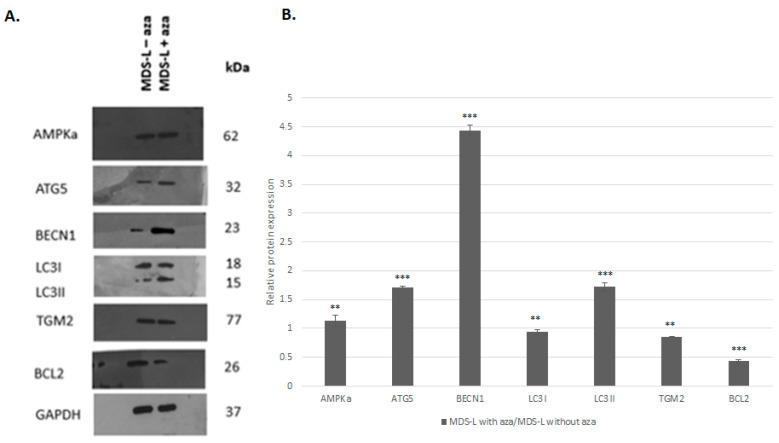
Azacitidine treatment can promote the activation of autophagy by upregulating promoting proteins and downregulating inhibitory proteins. (**A**) The immunoblot analysis of the expression of a series of proteins implicated in the processes of autophagy and apoptosis in MDS-L cells with and without treatment with azacitidine (MDS-L with aza and MDS-L without aza, respectively). Overall, azacitidine treatment was associated with higher expression levels of the AMPKA, ATG5, BECN1, and LC3II proteins, as well as lower expression levels of BCL2. (**B**) A bar graph depicting the relative expression levels of all proteins in the treated MDS-L cells compared to those in the baseline (untreated) MDS-L cells. Asterisks represent statistically significant differences; one asterisk (*) corresponds to *p* < 0.05, two asterisks (**) correspond to *p* < 0.02, and three asterisks (***) correspond to *p* < 0.001. Error bars correspond to standard errors (SEs).

To validate these findings, we assessed the effect of azacitidine treatment on critical proteins, namely AMPKα, ATG5, BECN1, LC3I/II, TGM2, and BCL2 (Figure 4). In detail, azacitidine treatment led to a statistically significant increase in AMPKα, ATG5, BECN1, and LC3II. Conversely, the TGM2 and BCL2 levels decreased significantly 1.2-fold and 2-fold after the azacitidine treatment.

Of importance, the effect of the azacitidine treatment on the expression of relevant genes at the mRNA and protein levels was highly consistent for *ATG5*, *BECN1*, *LC3II*, and *BCL2* (Table 2). *TGM2* was the only gene that exhibited an unaffected state at the mRNA level, accompanied by a significant decrease at the protein level.

### 3.4. Azacitidine May Also Affect Phosphoproteins Related to Autophagy and Apoptosis

Finally, we investigated the effects of azacitidine by analyzing the expression of 21 relevant phosphoproteins using an ELISA. Table 3 depicts the effect of azacitidine on the phosphoprotein expression levels in the MDS-L cells compared to those in the untreated MDS-L cells. Our results indicate that azacitidine significantly modulated the majority of these key signaling proteins (12/21, 57.1%), yet with different effects.

Specifically, CHK2, c-JUN, ERK1, and P53 displayed a statistically significant increase in their expression patterns. In contrast, azacitidine treatment resulted in a significant decrease in the expression levels of eight key regulators of pro-survival pathways, namely AKT, CREB1, EGFR, MARCKS, mTOR, NFKB, RSK1, and STAT3. The phosphorylation status of the remaining nine phosphoproteins was unaffected by azacitidine treatment, indicating a level of specificity.

## 4. Discussion

The pathogenesis of MDS is a complex and multifactorial process, involving deregulation in various cellular pathways, including autophagy [5,6] and apoptosis [8]. We recently demonstrated the significant downregulation of genes implicated in autophagy and apoptosis in MDS, which was more evident in HR-MDS patients [12]. Here, we validated this notion by examining the status of relevant proteins, as well as the effect of azacitidine [16] on their regulation. Azacitidine is a hypomethylating agent primarily recommended for HR-MDS patients who are not eligible for intensive therapies, as well as LR-MDS patients who have significant cytopenia or anemia [18]. Overall, we report the downregulation of autophagy- and apoptosis-related proteins in MDS compared to healthy individuals, with this phenomenon being more evident in HR-MDS patients. This finding further corroborates our previous claim [12] that the deregulation of these processes becomes more pronounced and thus relevant as the disease progresses.

Focusing on autophagy, our findings indicate the widespread downregulation of ATG proteins in MDS, namely ATG5, ATG12, and ATG16. This is consistent with previous research in a murine model of myelodysplasia [19] showing a significant decrease in the expression of p-ATG1, p-ATG6, ATG7, and ATG12. We also observed the significant downregulation of key autophagy regulators such as PI3KC3 and AMBRA1. Of relevance, PI3K deletion promoted myelodysplasia in a triple knockout (TKO) mouse model [20]. In the same context, CD34^+^ cells were characterized by downregulation of the PI3K signaling expression signature in MDS patients compared to that in healthy donors [21]. On the other hand, AMBRA1 has been shown, under mTOR inhibition, to lead to a reduction in the cell division rate [22]; of relevance, we observed that azacitidine treatment led to significant downregulation of the mTOR protein and the upregulation of AMBRA1 at the mRNA level.

Our findings are also in line with the impairment of apoptosis in MDS, as evidenced by the reduced expression of caspases 3, 7, and 8 in both MDS risk groups compared to that in the healthy donors. This downregulation suggests that MDS cells may evade programmed cell death, leading to increased survival of abnormal progenitor cells [23]. Of particular interest was the downregulation of caspase 8, a master regulator of PANOptosis, a form of cell death that is highly relevant in cancer [24]. Moreover, the observation of reduced caspase 3 activity specifically in MDS progenitor cells indicates cell-type-specific apoptosis resistance [25]. Furthermore, a study in *CASP8* knockout mice demonstrated that CASP8 deficiency results in an MDS-like phenotype [26].

Finally, our study showed the upregulation of TGM2 in both MDS risk groups, highlighting both its relevance to MDS pathogenesis by dysregulating autophagy and apoptosis and its potential role as a therapeutic target [27], as we previously reported [12]. A possible explanation for this upregulation is that TGM2 stabilizes pro-apoptotic BAX in an inactive conformation, thereby suppressing apoptosis [28]. Additionally, TGM2’s activity can promote autophagosome formation under stress, serving as a compensatory survival mechanism when the canonical autophagy pathways (e.g., the ATG5-ATG12-ATG16 axis) are impaired [29]. In line with this, BCL2 overexpression may inhibit both autophagy and apoptosis, highlighting its potential role—alongside TGM2—as a therapeutic target in MDS [12,30]. Upregulation of LC3I was also found in both MDS risk groups, which could reflect autophagy arrest at the lysosomal stage. Of relevance, studies in PI3K-deficient hematopoietic stem cells (HSCs) revealed reduced autophagic flux due to impaired lysosomal degradation, leading to LC3I buildup despite decreased LC3II [20].

Altogether, the general downregulation of both autophagic and apoptotic processes suggests the profound impairment of cellular homeostasis in MDS [31,32], allowing malignant cells to evade both programmed cell death and quality control mechanisms. Notably, previous studies have highlighted that defective autophagy can impair apoptotic signaling and vice versa, further supporting the notion that coordinated regulation of these pathways is essential for the prevention of malignant transformation [33]. Thus, our findings underscore the importance of restoring both autophagy and apoptosis to re-establish cellular equilibrium and suggest that therapeutic strategies targeting both processes may offer enhanced clinical benefit in MDS.

The second aim of our project was to investigate the impact of azacitidine on the processes of autophagy and apoptosis. In this context, we utilized the MDS-L cell line, which is considered a valuable model for testing therapeutic targets like azacitidine [34]. Overall, treatment with azacitidine led to restoration in the expression of autophagy- and apoptosis-related proteins in the MDS-L cells.

In terms of autophagy, we observed significant upregulation of a series of genes, including *ATG5*, *BECN1*, *CTSB*, *DRAM1*, and *LC3II*, among others, further supported by the upregulation of the ATG5, BECN1, and LC3I/II proteins. Of relevance, exposure of BM-derived mast cells (BMMCs) from MDS patients to azacitidine resulted in increased levels of ATG5, BECN1, and LC3II, indicating the induction of autophagy [35]. We also observed a significant decrease in the expression levels of AKT, mTOR, and RSK1, components of the PI3K/AKT/mTOR signaling pathway. This pathway acts as a negative regulator of autophagy in various diseases [36], frequently being hyperactivated in MDS and AML [37].

Regarding apoptosis, azacitidine treatment of the MDS-L cells resulted in a significant increase in the mRNA expression of *CASP3*, *CASP7*, and *CASP8*. Most notable was the tenfold increase in the *CASP7* gene, which is consistent with previous studies demonstrating CASP7 activation in the SKM1 [38] as well as the HL-60 and K562 cell lines [39] in response to treatment with decitabine, which shares a similar mechanism of action with azacitidine [40]. Another prominent effect of the azacitidine treatment was the significant suppression of BCL2, both at the mRNA and protein levels, which has previously been reported as a key mechanism underlying azacitidine-mediated cytotoxicity in various cancer cell lines (namely P39, HL60, and Jurkat) [41]. Mechanistically, this could be due to the reduced levels of NF-κB and CREB, as observed in the present and previous studies [42,43]. At the therapeutic level, the downregulation of BCL2 in the MDS-L cell line suggests that azacitidine may directly influence apoptosis. A first link for this interaction in MDS was provided through a significant association between azacitidine resistance and the percentage of malignant cells expressing BCL2L10 [44]. In contrast, the BCL2 expression levels were relatively stable, showing no significant differences between azacitidine-sensitive and azacitidine-resistant patients. This discrepancy may indicate that while azacitidine can effectively downregulate BCL2 in the MDS-L line, the response in primary patient samples may be influenced by additional factors, such as the presence of BCL2L10. Understanding these dynamics can help refine the therapeutic approaches, especially when combining azacitidine with BCL2 inhibitors like venetoclax in the treatment of AML and MDS [45].

Another effect of azacitidine treatment on apoptosis was evidenced through the upregulation of key proteins, including p53, CHK2, and JUN; this suggests that azacitidine may enhance apoptosis through the activation of DNA damage response pathways [46,47] and transcriptional regulation [48]. In particular, p53 activation may be triggered by the ERK pathway [49], which is known to play a complex role in cell survival, autophagy, and programmed cell death [50]. Relevant evidence in other types of cancer includes the ERK1-mediated phosphorylation of BCL2 and its subsequent removal by Beclin-1 leading to the promotion of autophagy in a human lung cancer cell line [49] and the enhancement in the activity of the MEK/ERK pathway after azacitidine treatment in gastric cancer [51].

A possible explanation for the combined effect of azacitidine on autophagy and apoptosis in the context of MDS is that it may induce cellular stress, leading to both processes being triggered, as has been reported in other hematological malignancies [52] and solid tumors [53]. Even though autophagy is generally considered an antagonist of apoptosis, prolonged or intense cellular stress may force autophagy to facilitate or coexist with apoptosis [11]. In fact, several molecules, such as BECN1 and CASP8, may serve as points of crosstalk between these two pathways, enabling a dynamic balance between survival and cell death [54]. Thus, the concurrent activation of autophagy and apoptosis observed in our experiments likely reflects the complex interplay between these processes in MDS cells under therapeutic pressure.

Our findings are also consistent with azacitidine having additional effects, as evidenced by the downregulation of EGFR and MARCKS, two proteins involved in cell proliferation [55,56]. The EGFR downregulation suggests that azacitidine may suppress MDS cell proliferation by inhibiting EGFR-mediated MAPK signaling [57], while the MARCKS downregulation may also prevent PI3K activation [58]. Finally, the azacitidine treatment led to a reduction in CREB1 levels, potentially suppressing its role in promoting cell proliferation and survival in AML and MDS [59,60,61]. Of note, CREB1 may enhance apoptosis, suggesting a mechanism for azacitidine’s anti-proliferative effects in CREB1-driven leukemias [62]. Along these lines, the decreased levels of p38 MAPK after the azacitidine treatment corroborates the finding that p38α silencing led to enhanced hematopoiesis in MDS BM progenitors in vitro [63].

Another critical effect of azacitidine involves the inhibition of NF-κB and STAT3 signaling, both of which are associated with inflammation and cell survival in hematological malignancies [64]. Constitutive activation of NF-κB is frequently observed in many cancers, and suppressing NF-κB limits the proliferation of cancer cells [65]. In this context, persistently activated STAT3 maintains constitutive NF-κB activity [66]. Moreover, STAT3 is significantly upregulated in HSPCs derived from MDS and AML patients, being associated with an increased percentage of blasts, an adverse prognosis, and lower overall survival in MDS [67].

Our study has possible limitations, such as the comparison between BM samples from MDS patients and PB samples from healthy donors. In this context, we recently showed that a comparative analysis of the mRNA expression of key autophagy-related genes in BM and PB samples from MDS patients revealed no significant differences, supporting the feasibility of using the latter in these analyses [12]. Furthermore, given the relatively small size of the present cohort, further validation of our findings in larger series of primary samples from MDS patients would be beneficial, especially regarding characterization of the effect of azacitidine on autophagy and apoptosis. Nevertheless, our research is pioneering in systematically analyzing the expression patterns of a sizeable fraction of proteins implicated in autophagy and apoptosis in MDS with different prognoses. We report evidence of the downregulation of these critical processes in MDS, particularly HR-MDS, and that azacitidine treatment may crucially affect both processes to restore cell homeostasis. These findings reinforce the efficacy of azacitidine in treating MDS and suggest that further exploration of combination therapies targeting critical molecular pathways could improve the outcomes for MDS patients.

## Figures and Tables

**Table 1:** The expression differences in the form of the fold changes in 15 proteins related to the processes of autophagy and apoptosis in all MDS patients, as well as in each MDS risk group, compared to a group of healthy donors. Statistically significant differences between any MDS group and the group of healthy donors are given in the form of *p*-values.

	All MDS	*p*-Value	LR-MDS	*p*-Value	HR-MDS	*p*-Value
**AMBRA1**	−1.190165		−1.042547		−1.361363	
**ATG5**	−3.274832	<0.001	−2.911969	<0.001	−3.066072	<0.001
**ATG12**	−1.774992	<0.001	−1.126027		−2.659288	<0.001
**ATG16**	−1.483484		−1.470517		−1.720712	
**CTSB**	−2.335516	<0.02	−2.318447	<0.05	−2.393512	<0.02
**DRAM1**	−1.916758	<0.02	−1.692008		−1.984616	<0.05
**LC3I**	1.612848		1.188060		1.664416	
**LC3II**	−3.231055	<0.001	−2.523108	<0.001	−4.274320	<0.001
**PI3KC3**	−1.509030		−1.478196		−1.566015	
**TGM2**	2.229709	<0.001	2.162048	<0.001	2.249299	<0.001
**UVRAG**	−1.307206		−1.042216		−1.797932	
**CASP3**	−2.650488	<0.001	−2.542133	<0.001	−2.799367	<0.001
**CASP7**	−2.410310	<0.001	−2.095823	<0.05	−3.116006	<0.001
**CASP8**	−2.013422	<0.05	−2.109531	<0.05	−1.961725	
**BCL2**	1.647007	<0.02	1.257670	<0.02	2.051875	<0.001

**Table 2:** Gene and protein expression differences (in the form of fold changes) in 6 proteins implicated in autophagy and apoptosis in MDS-L cells cultured in the presence of azacitidine compared to MDS-L cells without any type of treatment, which served as the controls. Asterisks (*) correspond to statistically significant differences (*p* ˂ 0.05).

Gene/Protein	Gene Expression	Protein Expression
AMPKα	-	1.32 *
ATG5	5.58 *	1.72 *
BECN1	4.03 *	4.44 *
LC3II	4.63 *	1.72 *
TGM2	1	−1.19 *
BCL2	−18.54 *	−2.28 *

**Table 3:** Expression differences (in the form of fold changes) in 21 phosphoproteins implicated in autophagy and apoptosis in MDS-L cells cultured in the presence of azacitidine compared to those in MDS-L cells without any type of treatment, which served as the controls. Asterisks correspond to statistically significant differences (*p* < 0.05).

Phosphoprotein	Fold Change
AKT	−2 *
AKT1S1	−1.25
CHK2	1.17 *
c-JUN	1.13 *
CREB1	−2.2 *
EGFR	−1.62 *
ERK1	1.76 *
FAK1	1.1
GSK3a/β	−1.15
HSPB1	−1.21
IKBA	1.18
MARCKS	−1.65 *
MEK1	1.2
MTOR	−1.2 *
NFKB	−1.97 *
P38 MAPK	−1.12
P53	2.3 *
PTN11	−1.21
RSK1	−1.16 *
SMAD3	−1.12
STAT3	−1.23 *

## Data Availability

The data presented in this study can be made available by the corresponding author on request due to privacy issues.

## Supplementary Materials

The following supporting information can be downloaded at https://www.mdpi.com/article/10.3390/cimb47070520/s1.
