# Supplementary material for: Restoration of Autophagy and Apoptosis in Myelodysplastic Syndromes: The Effect of Azacitidine in Disease Pathogenesis
Source: Curr Issues Mol Biol. 2025 Jul 4;47(7):520. doi: 10.3390/cimb47070520 (PMC12293780; doi:10.3390/cimb47070520)
Supplement: Supplementary file 1 [file cimb-47-00520-s001.zip › Tsekoura et al. Supplementary Material.pdf]

Materials and methods

Study Group

The study group comprised 20 MDS patients with a median age of 71 years (range: 59–82 years) and 14 healthy donors with a median age of 55 years (range: 24–72 years). All patients were diagnosed with MDS based on the 2016 World Health Organization (WHO) classification criteria and had not received treatment before sampling. Bone marrow samples were obtained from all patients included in the study, while peripheral blood (PB) samples were collected from all healthy controls.

In the present study, MDS patients were divided into two main groups: those with an IPSS-R score of ≤3.5 were classified into the lower-risk group (LR-MDS, n=12), whereas patients with an IPSS-R score of >3.5 were classified into the higher-risk group (HR-MDS, n=8).

MDS-L cell viability after azacitidine treatment

The MDS-L cell line was cultured as follows: cells were transferred to 96-well plates at a concentration of 20,000 cells per well and treated with azacitidine. A series of different azacitidine concentrations were applied, namely 0.5, 1.0, 3 and 5.0 µM within a controlled environment (37°C, 5% CO_2). Cells were harvested at different time points, namely 24, 48 and 72 hours, and assessed for cell viability using a specific assay using an isotonic solution of the Trypan Blue dye. Shortly after, alive and dead cells were counted using a hemocytometer. Each type of culture was performed three times, and the average values of alive and dead cells were used to determine the concentration of the drug that would induce effective cell apoptosis (IC50: half-maximal inhibitory concentration).

Gene expression analysis with quantitative Real-Time PCR

cDNA was synthesized using the Superscript II reverse transcriptase (Invitrogen, Carlsbad, CA, USA). PCR amplification was performed using the Hot Firepol EvaGreen Taq polymerase (Solis Biodyne, Tartu, Estonia) on a PikoReal^{TM} Real-Time PCR System (Thermo Fisher Scientific, MA,

USA). Gene expression levels were normalized against those of the *HPRT1* reference gene using the $2^{-\Delta\Delta CT}$ method. All relevant experiments were run in triplicates.

Protein expression analysis with Western Blotting

Twenty-five μg of proteins were separated via 7-12% SDS-PAGE and blotted onto PVDF membranes, which were blocked with 5% non-fat milk at room temperature for one hour. Subsequently, the membranes were incubated with specific primary antibodies overnight at 4°C. Blots were probed with secondary goat anti-mouse or goat anti-rabbit antibodies at room temperature for one hour, visualized using an ECL system (Thermo Fisher Scientific, MA, USA) and analyzed via Image J. Experiments were performed three times for each sample. To ensure equal protein loading between the paired treated and untreated samples, the presence of GAPDH protein was assessed.

Phosphoprotein expression analysis with a multiplex ELISA assay

MDS-L cells were lysed in Tris-HCl buffer containing protease and phosphatase inhibitors [ProtATonce lysis buffer (ProtATonce, Athens, Greece)], incubated at -20°C, thawed, sonicated, and centrifuged. The protein concentration of cell lysates was adjusted to 250 μg/μL using the Pierce™ BCA Protein Assay Kit (Thermo Fisher Scientific, MA, USA). xMAP assays were performed on a Luminex FLEXMAP 3D® platform (Luminex, Austin, TX), using a custom-developed phosphoprotein 21-plex panel (ProtATonce, Athens, Greece). Lysates were incubated with xMAP beads, followed by sequential incubation with detection antibodies and streptavidin-phycoerythrin. Beads were washed, resuspended in PBS-BSA, and analyzed. Phosphoprotein levels were assessed in three treated and three untreated samples; median bead signal intensities were determined for each phosphoprotein, and expression levels were calculated as the ratio of the median intensity in treated versus untreated samples, presented in the form of fold-change values.
